# Evaluating the impact of COVID‐19 on cancer declarations in Quebec, Canada

**DOI:** 10.1002/cam4.5389

**Published:** 2022-11-16

**Authors:** Agnihotram V. Ramanakumar, Bourassa Annie, Lamonde Frederic, Bertrand Christine, Rouleau Cathy, Latreille Jean

**Affiliations:** ^1^ Québec Tumor Registry Québec City QC Canada; ^2^ Research Institute‐McGill University Health Center Montréal QC Canada

**Keywords:** breast cancer, cancer diagnosis, colorectal cancer, COVID impact, COVID‐19, lung cancer, pandemic, prostate cancer, stage shift

## Abstract

**Background:**

COVID‐19 affected healthcare worldwide, limited access to healthcare, and delayed cancer screening and diagnosis. In this study, the effect of the first year of COVID‐19 was determined on cancer diagnoses in the province of Quebec, Canada.

**Methods:**

Data were collected from the 13 Quebec Cancer Registry health institutions. Newly diagnosed cancer declarations in the first year of the COVID‐19 (April 2020–March 2021) were compared with the reference periods (averages of 3 previous years). The main focus was on four leading cancers: lung, prostate, colorectal, and breast cancers. Generalized regression models with a poisson approximation and interrupted time series (ITS) analysis were used. Underestimated cases were presented in terms of relative risk (RR) and 95% confidence intervals (CI). The changes in the stage‐specific counts were also assessed in each of the four cancers. Results were illustrated separately for the first 4 months of the pandemic (first wave).

**Findings:**

This study estimated an overall under‐reporting of 15.3% (29,019 vs. 24,584) of declarations. This under‐reporting was evident across all age groups above 35 years (*p* < 0.0001), four primary cancers (*p* < 0.0001), all stages of cancers (*p* < 0.0001), and both sexes (*p* < 0.0001). Based on the relative risks, stage‐specific lung cancer counts were underestimated by 5%–34% in the first wave (0%–11% in the first year), prostate cancer by 16%–46% in the first wave (0%–25% in the first year), colorectal cancer 15%–45% in the first wave (0%–24% in the first year), and breast cancer 3%–45% in the first wave and (0%–28% in the first year). However, no stage‐IV cancers were statically under‐reported compared to the pre‐pandemic era and not even in the first wave.

**Interpretation:**

Cancer diagnosis was underestimated due to the COVID‐19 pandemic in the first year; this effect was more evident in the first phase of the pandemic in Quebec. Further research is required to determine the accurate burden of the disease in the long term.

## Graphical abstract

This study estimated an overall under‐reporting of 15.3% of Cancer declarations in the first year of the Pandemic in Quebec, Canada. This effect was more evident in the first phase.

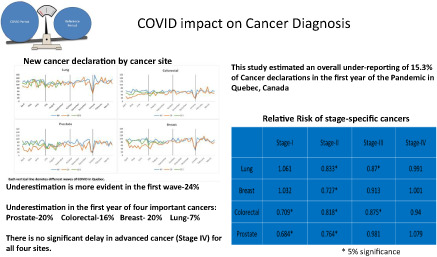

## 1 BACKGROUND

The COVID‐19 virus was first identified in Wuhan (Hubei Province), China, in late 2019 and rapidly spread around the globe. By March 11, 2020, the World Health Organization (WHO) declared it a global pandemic.^1^ The Province of Quebec (QC) was the most affected in Canada by COVID‐19‐related new cases and deaths, especially during the first wave of the pandemic (April–July 2020). The provincial government of Quebec introduced a 12–16 week lockdown from March 19, 2020, to minimize human‐to‐human viral transmissions and hospitalizations. The Government of Quebec also imposed several public health‐related restrictions to contain the pandemic.^2^

Health resources channeled their services toward COVID‐19 patient care, while many non‐COVID‐19‐related care activities, diagnoses, and therapeutic services were postponed or canceled globally and in the Province of Quebec. This situation had an immediate and anticipated impact on the clinical processes in Oncology, such as breast screening programs, colonoscopies, histopathology examinations of biopsies, and radiological imaging services such as computed tomography (CT) scans, magnetic resonance imaging (MRI) scans, and Positron Emission Tomography (PET) scan, which is all instrumental in detecting new cancers. Despite efforts to maintain a level of care in the health services sector, cancer care departments experimented with substantial difficulties in diagnosing, managing, and maintaining the new cancer case volumes worldwide and in Quebec.^3^, ^4^, ^5^ Recent studies have pointed out a remarkable reduction of new cancer diagnoses in Europe and the USA during the pandemic.^6^, ^7^ This pandemic‐related a decrease in access to care that may have delayed the identification of new cancers, resulting in worse long‐term outcomes, such as the expected emergence of more advanced stage cases and the worst prognosis.^8^ The significant delays in diagnosis and access to treatment may result in more advanced cancers and may lead to increased mortality.^8^, ^9^, ^10^

Though there was a gradual lifting of public health restrictions in the Summer of 2020, the recurrent COVID outbreaks, the emergence of new variants, and a backlog of procedures coupled with patient fear of COVID exposure, health care visits, and procedures were kept well below historical levels across the Province of Quebec.^4^ This accumulated a backlog of the health system with undiagnosed cancers due to the COVID‐19 pandemic, which has not been well established in real‐world settings. However, some efforts compare the number of surgeries, radiotherapy outcomes, pathology reports, colonoscopies, and breast imaging results.^11^ The publicly funded health system provides systematic population‐level surveillance data that includes a broad range of information on cancer sites, gender, and age distribution across the Province of Quebec. Given the standardized care practice in Quebec, the number of new cancer declarations provides a proxy estimation for the incidence of cancer volume. The changes between pre and post‐pandemic periods impact the healthcare system's pandemic‐related burden.

Four primary cancers represent the leading cause of cancer‐related deaths in Canada and Quebec Province. They also represent more than half of the cancer burdens each year.^12^ These are the primary cancers, such as prostate, colorectal, breast, and lung cancer, which account for a significant proportion of all clinical cancer burdens. Lung cancer has a clinical spectrum and potential overlap of symptoms and radiological findings with COVID‐19 disease.^13^ Hence, knowing the differentials in diagnosis may be necessary to understand the future challenges in cancer care. Another essential consequence of all delays is the changing profile of the cancer stage at diagnosis in these leading cancers and their effects on cancer mortality. Recent studies discussed the delays in the new cancer diagnoses^14^, ^15^ and their impact on the stage shift toward advanced malignancies and the worst survival rate.

In this paper, the impact of the pandemic is determined in the Province of Quebec on the stage of cancer at the time of declarations for four primary cancer sites and, for all cancer sites, their differentials by age, sex, and geography. The number of new cancer declarations was estimated during the pandemic (April 2020–March 2021) compared with the previous 3 years (April 1st 2017–March 31st 2020). Possible pattern shifts were observed to plan for resources effectively.

## 2 METHODS

This study examines new declarations of cancer reported by the contributing health institutions of the Quebec Cancer Registry (QCR). QCR invited all participating centers to be part of this validation exercise; however, only 13 centers out of 24 could contribute the data within the time frame due to a lack of resources while coping post‐COVID scenario. The data represent nearly 60% Quebec Cancer Registry catchment area. There is an opportunity to examine the age, stage, and sex‐specific variations in cancer cases across Quebec. There are no health disparities and healthcare access issues in Quebec due to universal healthcare programs. New cancer declarations were considered as a proxy for cancer incidence. All patients with a new cancer declaration identified in the selected healthcare facilities from April 1, 2017, to March 31, 2021, were evaluated. The health centers follow the standard quality check, such as coherence between incidence dates and date of birth, cross‐validating age with topography/morphology, cross‐checking behaviors/grades/laterality, and implementing standard multiple primary definitions. Finally, a comparison exercise has been conducted to compare the declarations with incident cases using 2017 registry data. It reveals that we can use declarations as a proxy of incidence cases as they were very similar in completeness (declarations have 95% incidence cases). No particular Ethical Review is required for this study. The general rules are applied to Quebec Cancer Registry, which collects the secondary data from the contributing institutions. This study is done to meet the mandate of the Ministry of Health and Social Services, Quebec. No human tissues are involved in this study.

Patients from all cancer sites were included to assess the difference in the number of new cancer declarations. All recent declarations of cancer were collected according to the registry standards. These standards are the same for all the participating centers across the understudy between 2018–2019, enabling the comparison before and after the pandemic era. The impact of coding standards in 2017 was evaluated, and the difference was insignificant in numbers. A detailed list of case identification codes is available in Appendix S2.

This study defined the 3 years before the COVID era as a ‘reference period’ (RP). This group considered new cancer declarations from April 1, 2017, to March 31, 2020 (two centers provided only 2 years during RP). For the ‘COVID‐19 period’ (CP), the data were from April 1, 2020, to March 31, 2021. After standardizing them to a calendar year of 52 weeks, the number of declarations was grouped by weeks. The counts were averaged each week to the totals of the consecutive years under study (3 years for the reference period); the week was treated as a unit of analysis in our regression models. Simple imputation was carried out for the last 2 weeks in December 2020 (due to Christmas) to address the issue of outliers in the statistical models. The difference between the two periods was taken as the raw deficit, and standardized percentage differences were calculated. If the difference was ≥0.1, it was considered significant. Chi‐square statistics were also used to determine the difference between the two periods (RP vs. CP). In addition, a similar exercise was repeated to see the differences in various characteristics such as gender (male, female), age group (<35, 35–49, 50–64, 65–74, 75+), type of cancer (4 primary cancers) and stage of cancer (I‐IV).

Generalized linear regression models were used with Poisson approximation^16^ to evaluate the under‐diagnosis of weekly cancer counts due to the pandemic (or backlog for the system). These models estimated the drop in the CP due to the pandemic relative to the cancer volume in the RP. Relative Risk (RR), 95% confidence intervals, and *p*‐values at 5% significance were used to determine the difference between the two periods (RP vs. CP). Assessing the impact in the first wave of the COVID onset was critical; hence, results from the first wave of COVID (April–July, 2020) have been presented separately.

To assess the stage‐specific profiles and learn more about the pandemic's overall impact, Interrupted Time‐Series (ITS) analysis^17^ was performed to evaluate the shift in the trend of the cancer declarations between RP versus CP. For this exercise, all the data from April 1, 2017, until March 31, 2021, were considered in consecutive weeks. After testing the autocorrelation function and residuals, ordinary least squares interrupted time series regression was conducted for the counts. Then, two regression slopes were compared (Beta‐1, the slope of the Pre‐COVID period; Beta‐2, the slope of the COVID period), and the pandemic's impact was presented in terms of the difference between the expected and observed number of COVID counts. The *p*‐values were evaluated at 95% confidence intervals to determine the difference between (Beta‐1 and Beta‐2) to determine the difference in trends. The statistical analysis was carried out by statistical software SAS 9.4.

## 3 RESULTS

**FIGURE 1:**
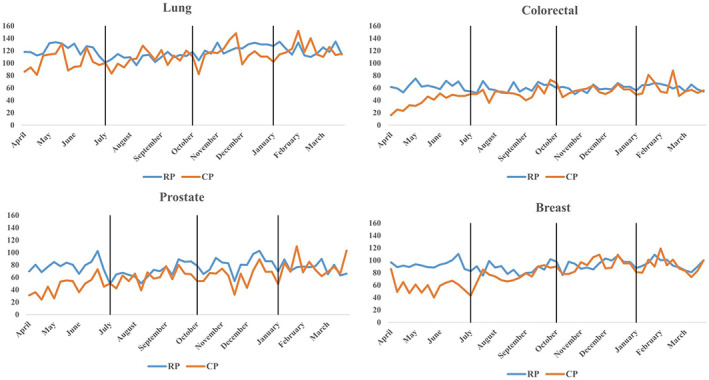
New declarations by cancer sites during reference and COVID Periods. Each vertical line denotes different waves of COVID Pandemic in Quebec

A detailed list of cancer sites was compared, and their newly declared cancer numbers between the reference and COVID periods are presented in Table 1. From April 1, 2017, to March 31, 2020, an annual average of 29,019 new cancer declarations were registered in the participating centers. A total of 15.3% of new cancer declarations were left behind during the pandemic (*n* = 24,584; *p* = 0.002) compared to the pre‐pandemic era. Table 1 reveals that the declarations of all cancer were not adequately reported except for larynx, non‐Hodgkin's lymphoma, ovarian, and testicle cancers. Three out of four primary cancers experienced a significant reduction in their numbers (*p* < 0.0001), i.e., breast (17.4%), colorectal (20.5%), and prostate (23.3%), whereas lung cancer only experienced a marginal reduction (7.7% *p* = 0.0526). The monthly distribution of declarations in both periods was presented in Figure‐1; it is clear from the contrast of these graphs that the significant impact happened during the first wave (April–July) of the pandemic in all four major cancers, and the gaps were narrowing as time progressed.

**TABLE 1:** Distribution of new cancer declarations in the participating institutions of Quebec

Cancer site	Reference period (RP)	COVID period (CP)	Standardized difference (%)
Lung	6046	5581	**−7.7**
Breast	4649	3842	**−17.4**
Prostate	3819	2929	**−23.3**
Colorectal	3080	2448	**−20.5**
Bladder	1061	864	**−18.6**
Non‐Hodgkin lymphoma	989	924	**−6.5**
Pancreas	802	700	**−12.7**
Uterus	806	731	**−9.3**
Melanoma	795	674	**−15.3**
Mouth	815	670	**−17.8**
Thyroid	805	625	**−22.4**
Kidney	677	582	**−14.0**
Stomach	398	308	**−22.6**
Liver	347	273	**−21.3**
Ovary	284	274	**−3.4**
Multiple myeloma	282	258	**−8.5**
Brain	321	262	**−18.4**
Leukemia	378	278	**−26.5**
Esophagus	254	212	**−16.6**
Larynx	163	163	**−0.2**
Cervix	196	159	**−18.7**
Hodgkin lymphoma	108	118	**9.3**
Testicle	58	71	**22.4**
Autres cancers	1885	1638	**−13.1**
TOTAL	29,019	24,584	**−15.3**

*Note*: Reference Period: April 2017–March 2020. COVID Period: April 2020–March 2021. Standard difference = (Proportion of RP.‐Proportion of CP)/Proportion of RP. A difference of ≥0.1 will be treated as a significant difference. The overall difference of total number of cancers is statistically significant (chi‐square *p* = 0.002). All the differences between RP and CP at 5% statistical significance are in the bold, otherwise non‐significant.

Patient characteristics of the four major cancer sites were analyzed in Table 2; patients had a median age of 68 years and an equal number of men and women, i.e., 50%. Cancer declarations were not adequately reported across all age groups, although the difference was insignificant in the early age group due to fewer patients (<35 years; 2.1% vs. 2.4%; *p* = 0.1887). The same level of underreporting was noticed across all cancer stages and in both sexes. The gap in the first wave was 17%–32% for age groups (6%–18% in the first year), about 26% for both men and women (12%–13% in the first year), and 13%–32% (3%–19% in the first year) for different stages of cancer.

**TABLE 2:** Distribution of new declarations and GLM estimates from the Poisson Regression

	Distribution of new cancer declarations			GLM Poisson Estimates		GLM Poisson Estimates first wave	
Characteristics	Reference period (RP)	COVID period (CP)	Standardized difference (%)	RR (RP vs. CP)	95% CI	RR (RP vs. CP)	95% CI
Age group							
<35 years old	635	585	−7.87	0.94	(0.84–1.05)	0.829	(0.68–1.01)
35 to 50 years old	2013	1741	−13.51	**0.896**	(0.84–0.95)	**0.777**	(0.69–0.87)
50 to 65 years old	8690	6772	−22.07	**0.812**	(0.79–0.84)	**0.679**	(0.64–0.72)
65 to 75 years old	9635	8257	**−14.3**	**0.886**	(0.86–0.91)	**0.733**	(0.70–0.77)
>75 years old	7817	6957	**−11**	**0.937**	(0.91–0.97)	**0.781**	(0.74–0.83)
Sex							
Male	14,568	12,186	**−16.35**	**0.873**	(0.85–0.89)	**0.733**	(0.70–0.76)
Female	14,591	12,407	−**14.97**	**0.884**	(0.86–0.90)	**0.735**	(0.70–0.77)
Cancer sites							
Lung	6076	5691	**−6.34**	**0.934**	(0.90–0.97)	**0.850**	(0.80–0.91)
Breast	4676	4004	**−14.37**	**0.860**	(0.83–0.90)	**0.668**	(0.62–0.72)
Colorectal	3101	2596	**−16.29**	**0.843**	(0.80–0.89)	**0.662**	(0.60–0.73)
Prostate	3842	3072	−**20.04**	**0.800**	(0.76–0.84)	**0.644**	(0.59–0.70)
Stages							
Stage 1	5979	5594	−**6.44**	**0.966**	(0.93–1.00)	0.770	(0.72–0.82)
Stage 2	3355	2591	−**22.76**	**0.801**	(0.76–0.84)	**0.678**	(0.62–0.74)
Stage 3	2634	2316	**−12.08**	**0.902**	(0.85–0.95)	**0.701**	(0.63–0.77)
Stage 4	3436	3337	**−2.87**	1.002	(0.96–1.05)	**0.870**	(0.80–0.94)

*Note*: A difference of more than 0.1 will be treated as a significant difference. All cancers with significance difference were denoted by bold. Reference Period: April 2017–March 2020. COVID Period: April 2020–March 2021. First Wave = April 2020–July 2020. RR = Relative Risk.

The availability of cancer stage information from the contributing centers is the same as in the pre‐COVID era in completeness (*p* = 0.4782). The cancer stage distribution of CP was compared with the RP and presented in Table 3. Overall, it showed significantly lower counts of Stage‐I and Stage‐II for prostate cancer and colorectal cancers (*p* < 0.0001), Stage‐III for lung cancer and colorectal cancers (*p* < 0.0001), and Stage II for breast cancer (<0.0001). However, there were no observations of any under‐reporting in all four cancers' at advanced stages (Stage‐IV). These results were cross‐validated with ITS analysis and presented in Appendix S1. This table consists of the expected and observed weekly counts illustrating the impact in real terms; the results are precisely the same as explained by GLM models to concrete these findings.

**TABLE 3:** Distribution of new declarations by cancer site and GLM estimates from the Poisson Regression

	Distribution of new cancer declarations			GLM Poisson estimates		GLM Poisson estimates first wave	
Characteristics	Reference period (RP)	COVID period (CP)	Standardized difference (%)	RR (RP vs. CP)	95% CI	RR (RP vs. CP)	95% CI
Lung							
Stage 1	1848	1950	5.50	1.064	(1.00–1.13)	0.949	(0.85–1.06)
Stage 2	479	419	**−12.59**	**0.885**	(0.78–1.01)	0.928	(0.75–1.15)
Stage 3	898	785	**−12.62**	**0.881**	(0.80–0.96)	**0.655**	(0.55–0.78)
Stage 4	1788	1758	−1.68	0.999	(0.94–1.07)	**0.930**	(0.83–1.04)
Prostate							
Stage 1	492	330	**−32.97**	**0.75**	(0.66–0.86)	**0.535**	(0.41–0.69)
Stage 2	1287	922	**−28.34**	**0.754**	(0.69–0.82)	**0.583**	(0.50–0.68)
Stage 3	480	447	−6.81	0.942	(0.83–1.07)	0.810	(0.65–1.01)
Stage 4	399	411	3.09	1.078	(0.94–1.23)	0.832	(0.65–1.06)
Colorectal							
Stage 1	525	373	**−28.91**	0 .**76**	(0.67–0.86)	**0.545**	(0.43–0.69)
Stage 2	606	475	**−21.66**	**0.828**	(0.74–0.93)	**0.717**	(0.58–0.88)
Stage 3	705	598	**−15.18**	**0.876**	(0.79–0.97)	**0.651**	(0.53–0.79)
Stage 4	566	551	−2.71	1.037	(0.92–1.16)	**0.853**	(0.70–1.04)
Breast							
Stage 1	2516	2458	−2.29	1.012	(0.96–1.07)	**0.783**	(0.71–0.87)
Stage 2	748	523	**−30.08**	**0.717**	(0.64–0.80)	**0.551**	(0.45–0.67)
Stage 3	305	252	**−17.47**	**0.907**	(0.77–1.07)	0.746	(0.56–0.99)
Stage 4	234	228	−2.70	1.031	(0.86–1.23)	0.970	(0.71–1.32)

*Note*: A difference of >0.1 will be treated as a significant difference. All the differences between RP and CP at 5% statistical significance are in the bold, otherwise non‐significant. Reference Period: April 2017–March 2020. COVID Period: April 2020– March 2021. First Wave = April 2020–July 2020. Standard difference = (Proportion of RP ‐ Proportion of CP)/ Proportion of RP RR = Relative Risk.

Paradoxically, the counts in Stage‐I lung and stage‐I breast cancer are more in the CP than in the RP. When the underreporting of counts in the first wave of the pandemic was observed; 5%–34% were for lung cancer (0%–11% in the first year), 16%–46% for prostate cancer (0%–25% in the first year), 15%–45% for colorectal cancer (0%–24% in the first year), and 3%–45% (0%–28% in the first year) for breast cancer. However, and interestingly so, even during the first phase of the pandemic, none of the cancers with advanced stage (IV) were statically under‐reported compared to the pre‐pandemic era.

The impact of the pandemic on the cancer stages is estimated in Table 4 using interrupted time‐series analysis. These results enable the understanding of the stage shift during the pandemic. Whenever the slope changes the direction between two eras, this may be treated as a proxy of stage shift. There is a possible shift in stage‐III and stage‐IV of lung cancer (*p* < 0.0001); Stage‐I (*p* = 0.005) and stage‐ II of prostate cancer (*p* < 0.0001), Stage‐I‐III of colorectal cancers (*p* < 0.0001), and Stage‐II of breast cancer (*p* < 0.0001).

**TABLE 4:** Interrupted time‐series analysis to estimate the impact of COVID‐19 on the counts of cancer sites and their stages

Cancer site	Stage 1				Stage 2				Stage 3				Stage 4			
	Beta	95% CI		*p*‐value	Beta	95% CI		*p*‐value	Beta	95% CI		*p*‐value	Beta	95% CI		*p*‐value
Lung																
Stage of RP (β1)	0.09	−0.07	0.25	0.2717	−0.01	−0.06	0.05	0.771	−0.03	−0.1	0.04	0.4231	−0.59	−0.17	0.06	0.3072
Stage of CP (β2)	**0.54**	0.38	0.7	**<0.0001**	**0.05**	0	0.11	**0.0584**	0.28	0.21	0.35	**<0.0001**	**0.48**	0.36	0.59	**<0.0001**
Difference between β1 versus β2				**<0.0001**				0.1233				**<0.0001**				**<0.0001**
Prostate																
Stage of RP (β1)	−0.02	−0.09	0.04	0.5366	−0.05	−0.16	0.05	0.3198	0.03	−0.03	0.1	0.2892	0.01	−0.05	0.07	0.7338
Stage of CP (β2)	**0.15**	0.08	0.21	**<0.0001**	**0.39**	0.28	0.5	**<0.0001**	0.15	0.09	0.22	**<0.0001**	**0.15**	0.1	0.21	**<0.0001**
Difference between β1 versus β2				**0.0005**				**<0.0001**				**0.0072**				**0.0005**
Colorectal																
Stage of RP (β1)	−0.12	−0.07	0.05	0.6789	−0.05	−0.11	0	0.064	−0.03	−0.09	0.04	0.4299	0.01	−0.05	0.07	0.7569
Stage of CP (β2)	**0.14**	0.08	0.2	**<0.0001**	**0.14**	0.08	0.2	**<0.0001**	**0.24**	0.17	0.31	**<0.0001**	**0.17**	0.11	0.23	**<0.0001**
Difference between β1 versus β2				**<0.0001**				**<0.0001**				**<0.0001**				**0.0002**
Breast																
Stage of RP (β1)	0.18	−0.02	0.38	0.0708	−0.14	−0.21	−0.07	**<0.0001**	0	−0.04	0.04	0.9271	0	‐0.04	0.04	0.8188
Stage of CP (β2)	**0.86**	0.65	1.07	**<0.0001**	**0.19**	0.12	0.27	**<0.0001**	**0.07**	0.02	0.11	**0.0022**	**0.07**	0.03	0.12	**0.0009**
Difference between β1 versus β2				**<0.0001**				**<0.0001**				**0.0195**				**0.0085**

All the differences at 5% statistical significance are in bold.

The graphical illustration of all stages in each cancer was displayed in Appendix S3; the counts have not yet been returned to those observed levels before the pandemic as the slope returns to the pre‐COVID era. These graphs most likely demonstrate the shift in the stages between two periods, exclusively when they change the direction between two periods, and to what extent the catchup activity was on the backlog of procedures from the initial phases of the pandemic. Looking at the differential in the proportions over time suggests that the pandemic may have influenced a possible slight shift in the stages, which requires more details.

## 4 DISCUSSION

The COVID‐19 outbreak has changed the landscape of the healthcare domain and affected the face of cancer diagnosis and cancer care globally. Although the efforts made by the Care Managers and providers, such as telehealth and passive health checks, reach out to patients to prevent diagnostic delays and the feared ‘upstaging effects,’ the confrontational effects are still a matter of speculation and a great deal of uncertainty that may have implications in years to come.^18^, ^19^ Like the other populations, Quebec, one of Canada's most affected Provinces (Statscan‐COVID19 Data perspective; https://www.statcan.gc.ca/en/covid19), will need further analysis and a retrospective point of view over the coming months and years to fully understand the cancer‐specific care issues triggered due to delays in the diagnosis induced by the pandemic.

This study shows an overall decline of 15.3% in the new cancer declarations in the first year of the pandemic. However, nearly one‐in‐four cases is missing during the first wave (24%) between April–July, 2020. The most significant decreases (>20%) in cancer cases were noted for leukemia, prostate, stomach, thyroid, liver, and colorectal cancers; only a minimal decrease (<5%) was noticed for ovarian and larynx cancers. The cancer case volume was also underdiagnosed with most of the selected characteristics of patients during the COVID‐19 period.

This study agrees with the drop in the number of new incidence cases reported globally,^7^, ^20^, ^21^ and confirms a similar reduction in lung cancer cases (7.7%) as reported by other studies (6.9%).^20^ This decline in lung cancer is consistent with London et al.^21^ and a US study based on 20 institutions.^22^ The estimated percentage of breast, prostate, and colorectal cancers agrees with under‐reporting in other studies from other Provinces and populations.^23^, ^24^, ^25^ The trend analysis shows the recovery of counts in all four cancers after the first wave, indicating the effect of the alternative methods adopted by the cancer care network in Quebec to catch up with the backlog of diagnostic care.^26^ Quebec chose to continue the screening services and surgical wards soon after the first wave in 2020. A massive backlog of cases, lack of human resources, succeeding waves, and changing variants have influenced patients to abstain from these programs and services.

Considering the spectrum of lung cancer presentation at diagnosis has the potential to overlap with the COVID‐19 disease. The difference in drop between lung cancer and other cancers could be due to diagnosis similarity in the manifestation of COVID pneumonia and lung cancer on the respiratory tract. Many patients could be found with lung tumors when they got a CT scan to evaluate their lungs when they had symptoms of COVID. However, cancer screening is key to certain cancers, including colorectal and breast cancer. Temporary and complete closure of screening services such as breast mammograms during the first wave might have caused the underreporting of cases. For example, colorectal screening tests (FIT tests) decreased by 74% in the first wave in Quebec, from April to June 2020, compared to the same period in 2019(Ref 26). Provinces across Canada, such as Manitoba, and Ontario, have reported a significant reduction (54%–98%) of their screening cases during the first wave and reported substantial catchup activities after July 2020.^27^, ^28^, ^29^ Another reason for the decline in reported counts may be related to the health‐seeking behavior of the population or the overburdened health system to handle the backlog. Health care providers need to address patient concerns regarding the risk of COVID transmission and support the idea of early detection and the treatment of cancers. Some missing cases may also be attributed to COVID‐19‐related deaths.^30^ This study only covers a little over 12 months since the start of the pandemic; thus, missed cancer diagnoses would, in most cases, not yet have progressed to death since many cancers would not experience a mortality event within a year of diagnosis.

Some published studies have demonstrated an adverse impact on the survival of patients with delays in cancer care, such as radiotherapy and surgery.^31^, ^32^ Most of these simulation studies were based on the assumptions of the early pandemic period to show the effect of diagnostic delays on health outcomes. Although these modeling studies provide some oversite, real‐world evidence is lacking in Canada and other populations. Our study attempted to address the impact of pandemic‐specific diagnostic delays on cancer stage at diagnosis and estimated the weekly backlogs to plan future resources in Quebec. These data only cover 60% of the catchment area of Quebec. Still, it is justified that our population showed a similar distribution of the total cancer incidences at the Quebec Cancer Registry (*p* = 0.4483). Our data suggested prioritizing cancer diagnostic services such as Pathology, imaging, and surgical resection— and developing screening algorithms and guidelines to triage the pending cases toward clearing the backlog. During a pandemic, a functioning and agile public health system is essential to ensure that patients see Clinicians when they develop symptoms and access diagnostic or pathology services. Screening programs should remain accessible as much as possible during the other pandemic waves, and cancer care should be prioritized.

Diagnostic delays may advance the stage at diagnosis, which requires a thorough understanding of the changing patterns of the cancer stage. More lung cancers are noticeable in the early stages (I) of CP than in RP (36% vs. 40%, *p* < 0.0991) in table‐3, and a similar trend was observed with breast cancer (66% vs. 71%, *p* < 0.2345). Advanced Stage (IV) numbers remain the same for all cancers as no statistical differences were observed (*p*‐values are insignificant). Stages II/III counts in the CP might have experienced a slight decrement relative to the RP. This might have happened due to the catchup with diagnostic activities after the first wave.

It can be estimated that the cancer stage shift is approximate to the change in regression lines and their directions between two periods (RP vs. CP). To understand the pattern of cancer stage distributions in both periods (RP vs. CP), there was an attempt to use Interrupted Time‐series Analysis; by observing the difference between the regression slopes in the RP and during the CP across the four stages in each of the four cancers. This analysis was treated as exploratory until the shifts were validated with external data from other populations.

A few limitations in this study were related to the completeness of the data. The declarations among the non‐participating institutions are unknown, although the data are well represented in the number of annual cases and the population of the entire QCR. This information is based on the declarations for each participating center, not on the incidence cases, permitting some duplicates. It may not be a big concern as the same data were standardized via the same data collection methods and coding practices during RP and CP. The team has assessed all records in the COVID period, reviewed the files transferred over the reference years, and determined comprehensive data.

In some cases, the processes may have been delayed. However, they were not disrupted and would not likely change cancer ascertainment at the registry level. Some patients with undiagnosed cancers may have died of other causes (such as COVID‐19 or other comorbidities).

The COVID‐19 Pandemic has posed challenges to Patients with cancer, the cancer network, the registration systems, and Researchers. It will be vital to study the interactions between cancer diagnosis and COVID‐19 to better tailor the management of Patients and preparedness for cancer care. The data in this study came from the Province of Quebec's participating institutions, which reflected the first pandemic year. In conclusion, a Cancer diagnosis was underestimated due to the COVID‐19 pandemic in the first year; this effect was more evident in the first phase (wave) of the pandemic in Quebec. Further research is required to understand the changing dynamics of the COVID‐19 virus and access to the services to estimate the accurate burden of the disease in the long term.

## AUTHOR CONTRIBUTIONS

**VENKATA RAMANA Ramana AGNIHOTRAM:** Conceptualization (lead); formal analysis (lead); investigation (equal); methodology (lead). **Annie Bourassa:** Data curation (equal); investigation (equal); project administration (equal). **Frederic Lamonde:** Data curation (supporting); formal analysis (supporting); methodology (supporting). **Christine Bertrand:** Conceptualization (lead); funding acquisition (lead); project administration (lead). **Cathy Rouleau:** Investigation (supporting); project administration (equal). **Jean Latreille:** Conceptualization (equal); funding acquisition (lead); project administration (lead).

## FUNDING INFORMATION

Government of Quebec, Canada.

## Supporting information


Appendix S1–S4

## Data Availability

Data sharing is not applicable to this article as no new data were created or analyzed in this study.
