# Supplementary material for: Evaluating the impact of COVID‐19 on cancer declarations in Quebec, Canada
Source: Cancer Med. 2022 Nov 16;12(5):6260–9. doi: 10.1002/cam4.5389 (PMC10028061; doi:10.1002/cam4.5389)
Supplement: Supplementary file 1 — Appendix S1–S4 [file CAM4-12-6260-s001.docx]

**Appendix** **1**: Interrupted Time-series analysis to estimate the impact of COVID on the weekly backlog in cancer sites and their stages

|  |  | Stage shift due to pandemic | | | | |
| --- | --- | --- | --- | --- | --- | --- |
| Type | Stage | Expected No. of Declarations per Week | Estimated No. of Declarations per Week | Pecentage Decline in Cases (%) | 95%CI | P-vlaue |
| Lung | Stage 1 | 39.4 | 35,2 | −11,10% | (−0.8, 21.6) | 0.066 |
|  | Stage 2 | 9.2 | 8.9 | −3.10% | (−25.6, 25.2) | 0.813 |
|  | Stage 3 | 17.1 | 12.7 | −26.40% | (10.5, 39.5) | 0.002 |
|  | Stage 4 | 34.8 | 32.2 | −8% | (−5, 19.3) | 0.217 |
| Prostate | Stage 1 | 9.9 | 5.2 | −47.80% | (30.8, 60.7) | <0.001 |
|  | Stage 2 | 25.2 | 14.7 | −42.30% | (31.5, 51.4) | <0.001 |
|  | Stage 3 | 10.5 | 7.7 | −27.40% | (6.3, 43.7) | 0.014 |
|  | Stage 4 | 7.8 | 7 | −10.40% | (−18, 32) | 0.435 |
| Colorectal | Stage 1 | 11 | 6.4 | −42.10% | (24.6, 55.5) | <0.001 |
|  | Stage 2 | 11 | 8.5 | −23.10% | (1.9, 39.7) | 0.034 |
|  | Stage 3 | 14.1 | 9.2 | −35.60% | (19.6, 48.5) | <0.001 |
|  | Stage 4 | 10.3 | 8.8 | −15.40% | (−7.8, 33.6) | 0.176 |
| Breast | Stage 1 | 55.2 | 41.7 | −24.90% | (16.2, 32.8) | <0.001 |
|  | Stage 2 | 12.3 | 8.5 | −31.60% | (13.7, 45.8) | 0.001 |
|  | Stage 3 | 5.8 | 4.8 | −16.50% | (−16.2, 40) | 0.284 |
|  | Stage 4 | 4.3 | 4.1 | 7% | (−34.2, 35.6) | 0.698 |

**Appendix** **2**: Topography sites and histology types used for different cancer sites in the QCR

| Cancer Site | Topography Site (ICD-O3) | Histology Type |
| --- | --- | --- |
| Bladder (including in situ for incidence) | C67.x | [*](#TF2) |
| Brain | C70.x-C72.x | [*](#TF2) |
| Breast | C50.x | [*](#TF2) |
| Cervix | C53.x | [*](#TF2) |
| Colorectal | C18.x-C20.x et C26.0 | [*](#TF2) |
| Esophagus | C15.x | [*](#TF2) |
| Hodgkin lymphoma | [†](#TF1) | 9650–9653, 9655, 9659, 9663 |
| Kidney | C64.x et C65.x | [*](#TF2) |
| Larynx | C32.x | [*](#TF2) |
| Leukemia | [†](#TF1) | 9742, 9800, 9801, 9806–9809, 9820, 9826, 9831, 9833–9834, 9840, 9860–9861, 9863, 9865–9867, 9869–9876, 9891, 9895–9898, 9910–9911, 9920, 9930–9931, 9940, 9945–9946, 9948, 9963–9964 |
|  | C42.0, C42.1 | 9727, 9811–9818, 9823, 9827, 9837 |
| Liver | C22.0 | [*](#TF2) |
| Lung | C34.x | [*](#TF2) |
| Melanoma | C44.x | 8720-8790 |
| Mouth | C00.x-C14.x | [*](#TF2) |
| Multiple myeloma | [†](#TF1) | 9731, 9732, 9734 |
| Non-Hodgkin lymphoma | [†](#TF1) | 9590–9597, 9671, 9673, 9678–9680, 9687–9691, 9695, 9698–9702, 9705, 9708–9709, 9712, 9714, 9716–9719, 9724–9726, 9735, 9737–9738, 9761 |
|  | [†](#TF1) except C42.0, C42.1 | 9727, 9811–9818, 9823, 9827, 9837 |
| Ovary | C56.x | [*](#TF2) |
| Pancreas | C25.x | [*](#TF2) |
| Prostate | C61.x | [*](#TF2) |
| Stomach | C16.x | [*](#TF2) |
| Testis | C62.x | [*](#TF2) |
| Thyroid | C73.x | [*](#TF2) |
| Uterus | C54.x et C55.x | [*](#TF2) |
| All other cancers[**](#TF3) | All sites C00-C80 not listed above | [*](#TF2) |

^a^ All topography sites.

^b^ Histology types 9050–9055 (mesothelioma), 9140 (Kaposi sarcoma), and 9590–9992 (leukemia, lymphoma, and multiple myeloma) are excluded from other specific organ sites.

^c^ Non-melanoma skin cancer (neoplasms, NOS; epithelial neoplasms, NOS; and basal and squamous) are excluded.

ICD-O-3 refers to the *International Classification of Diseases for Oncology, Third Edition*.

NOS = not otherwise specified.

**Appendix** **3**: Interrupted time-series analysis of the site-specific trend of four cancer sites (Beta1:Slope of RP; Beta2:Slope of CP; Beta 3: Shift in the slope due to COVID

**Appendix** **4** Table-1.a: Distribution of demographic and clinical characteristics

|  | Distribution of New Cancer Declarations | | | |
| --- | --- | --- | --- | --- |
| Characteristics | Reference Period (RP) | COVID Period (CP) | Standardized Difference (%) | Significance |
| **Age Group:** |  |  |  |  |
| Less than 35 years old | 635 | 585 | −7.87 | NS |
| 35 to 50 years old | 2013 | 1741 | −13.51 | * |
| 50 to 65 years old | 8690 | 6772 | −22.07 | * |
| 65 to 75 years old | 9635 | 8257 | −14.3 | * |
| More than 75 years old | 7817 | 6957 | −11 | * |
| **Sex**: |  |  |  |  |
| Male | 14,568 | 12,186 | −16.35 | * |
| Female | 14,591 | 12,407 | −14.97 | * |
| **Cancer sites** |  |  |  |  |
| Lung | 6076 | 5691 | −6.34 | * |
| Breast | 4676 | 4004 | −14.37 | * |
| Colorectal | 3101 | 2596 | −16.29 | * |
| Prostate | 3842 | 3072 | −20.04 | * |
| **Stages:** |  |  |  |  |
| Stage 1 | 5979 | 5594 | −6.44 | ***** |
| Stage 2 | 3355 | 2591 | −22.76 | ***** |
| Stage 3 | 2634 | 2316 | −12.08 | ***** |
| Stage 4 | 3436 | 3337 | −2.87 | ***** |

A difference of more than 0.1 will be treated as a significant difference (*).

All cancer differences are significant except “NS”.

Reference Period: April 2017–March 2020

COVID Period: April 2020–March 2021.
